# An unusual cause of right heart dilatation

**DOI:** 10.1186/1532-429X-18-S1-T4

**Published:** 2016-01-27

**Authors:** Yiying Han, Bao Ru Leong, Yi-Hui Hung, Alicia Er, Yunyun Go, Tiong Keng Lim

**Affiliations:** National Heart Centre Singapore, Singapore, Singapore

## Background

**Figure 1:**
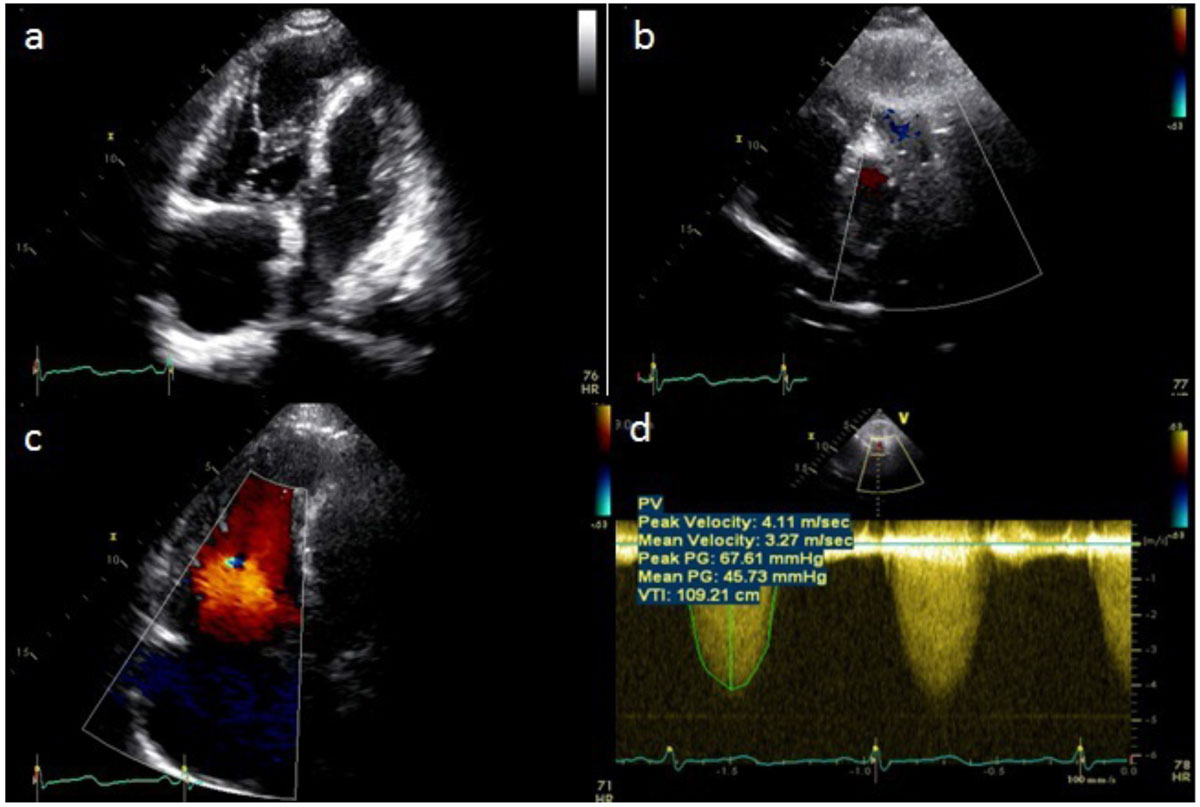
**a. 4-chamber view, b. RVOT view, c. 4-chamber with color Doppler, d. CW Doppler at RVOT**.

60 year-old lady with no significant medical history of note presented with shortness of breath on exertion for the past 3 months. Physical examination revealed a loud ejection systolic murmur at the left sternal edge. A transthoracic echocardiogram showed turbulent flow and increased velocity at the right ventricular outflow tract (RVOT). The right atrium and ventricle were dilated and there was severe tricuspid regurgitation (Figure 1). A cardiac MRI was ordered to ascertain the cause of RVOT obstruction.

## Results

There is a large mass causing near occlusion of the main PA. This mass extends distally into proximal left and right PA. This mass also extends proximally into pulmonary valve and proximal posterior aspect of the RV out flow tract.

**Figure 2:**
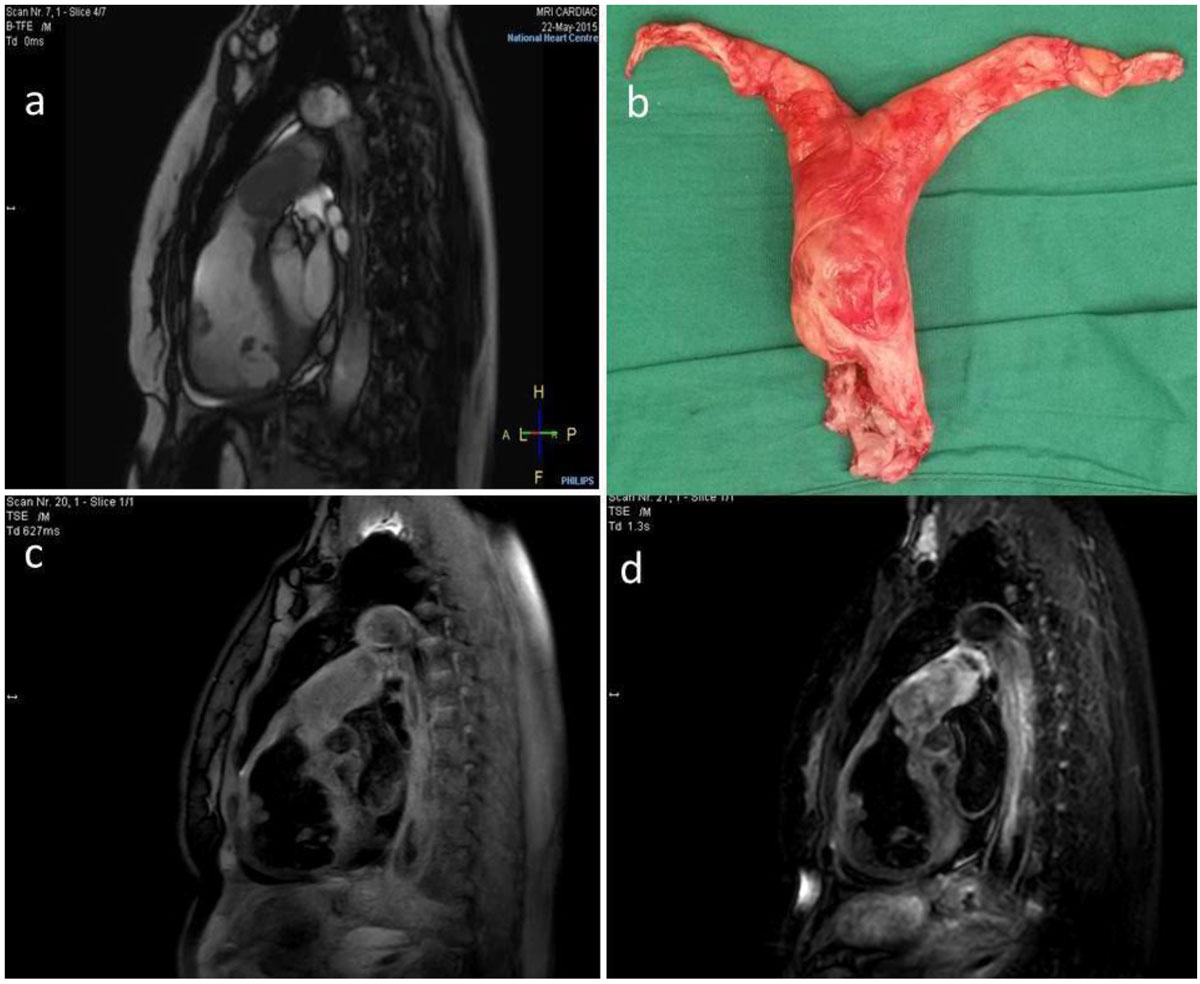
**a. RVOT view, b. Specimen showed PA sarcoma resected en-bloc, c. T1 TSE fat sat, d. T2 TSE STIR**.

On T1W images, the tumor shows heterogeneous low-signal while on T2W sequences, it exhibits a mixed signal with hyperintensity as well as areas of intermediate to low intensity (Figure 2). The lack of T1 hyperintensity would be against a diagnosis of thrombus.

## Conclusions

The patient underwent complete endarterectomy, pulmonary valve replacement and PA reconstruction using a bifurcated Gelweave graft. She recovered well and was discharged home subsequently. Histology showed intimal sarcoma with focal myofibroblastic differentiation.

Pulmonary artery sarcomas are exceedingly rare. Other important and more common differential diagnoses to be considered include pulmonary embolism (PE) or tumor embolism from pulmonary or other malignancies. Pulmonary artery sarcoma mimicking as PE, pulmonary artery aneurysm or Takayasu's disease has been reported in the literature. In our case, the absence of thromboembolism risk factors, together with heterogeneous tumor enhancement and invasion into the wall of main pulmonary artery as well as its branches are suggestive of a malignant process instead of PE.

